# Essential Oil Composition of *Bupleurum praealtum* and *Bupleurum affine*: New Natural Constituents

**DOI:** 10.3390/plants13152076

**Published:** 2024-07-26

**Authors:** Milica D. Nešić, Milan S. Nešić, Milan Ž. Dimitrijević, Niko S. Radulović

**Affiliations:** Department of Chemistry, Faculty of Sciences and Mathematics, University of Niš, Višegradska 33, 18000 Niš, Serbia; milica.stevanovic@pmf.edu.rs (M.D.N.); milan.nesic@pmf.edu.rs (M.S.N.); milan.dimitrijevic@pmf.edu.rs (M.Ž.D.)

**Keywords:** plant volatiles, *Bupleurum*, perillyl esters, NMR, spectral simulation, isomeric praealtaesters, 4-alkyl acetate

## Abstract

This study explores the chemical composition of essential oils from two Serbian *Bupleurum* species (Apiaceae), *Bupleurum praealtum* L. and *Bupleurum affine* L., traditionally recognized in Chinese medicine for their therapeutic potential but less studied for their essential oils. Through GC-MS analysis, we identified 230 constituents, revealing distinct profiles between the species. Perillyl 2-methylbutanoate was identified in *B. affine* oil for the first time, confirmed using synthetic approaches and characterized by advanced spectroscopic techniques, including two-dimensional NMR and spin-simulation of ^1^H NMR spectra. Additionally, new natural compounds, including tentatively identified 4-decyl acetate and 4-undecyl acetate, were discovered. The study also reports five stereoisomeric esters of tetradeca-5,7,9,11-tetraen-1-ol. These findings significantly contribute to the understanding of the phytochemical diversity within the genus *Bupleurum* and underscore potential differences in ecological adaptations or biosynthetic pathways among species.

## 1. Introduction

The genus *Bupleurum* L. (Apiaceae) encompasses a diverse array of plant species known for their aromatic and medicinal properties, and it is almost exclusively native to Europe and eastern Asia [1]. Species of this genus are well-known for their over 2000-year long usage in traditional Chinese medicine as “liver tonics”, for the treatment of fever-producing infections, common cold, inflammatory disorders, hepatitis, etc. [2,3] *Radix Bupleuri* is the most frequently mentioned ingredient of these preparations, and is derived from the dried roots of *Bupleurum chinense* DC. and *Bupleurum scorzonerifolium* Willd., although many other *Bupleurum* species are also used under the same name (*Bupleurum falcatum* L. and *Bupleurum yinchowense* R.H.Shan and Yin Li). It has been found to possess anti-inflammatory [3], antiviral [4], antidepressant [5], antitumor [6], hepatoprotective [7], and immunoregulatory activities [8].

The chemical composition of plant species belonging to this genus has been extensively studied. Triterpene saikosaponins are the primary active constituents of these plants, responsible for a broad spectrum of pharmacological activities in preparations containing *Radix Bupleuri* [1]. Polyacetylenes constitute a significant group of compounds found in plants of the Apiaceae family, exhibiting anti-inflammatory, antibacterial, anticancer, and antifungal properties, although some have been identified as toxic [9]. *Bupleurum longiradiatum*, widely distributed in northeastern China and available in certain herb markets, is a toxic plant primarily containing the toxic polyacetylenes: bupleurotoxin and acetylbupleurotoxin, compounds absent in other *Bupleurum* species [9]. Therefore, the polyacetylene profile can serve as a distinguishing feature among species within this genus.

Essential oils have gained attention for their diverse chemical compositions and therapeutic potential. The analysis of essential oils not only provides insights into the phytochemical profile of plants but also unlocks novel avenues for pharmacological exploration. Essential oils derived from the *Bupleurum* species have received comparatively less attention in scientific research. To date, essential oils from forty plant species within the genus *Bupleurum* have been chemically analyzed, with ten classified as annual and thirty as perennial species [10]. Li and colleagues conducted a study focusing on ten *Bupleurum* species originating from China, revealing that the predominant constituents were aliphatic aldehydes and acids such as hexanol, heptanol, heptanoic acid, octanoic acid, and hexadecanoic acid [11]. In addition to these, typical for Chinese species, the dominant compounds in the essential oil of *B. marginatum* were *β*-caryophyllene, *β*-caryophyllene oxide, and spathulenol [12]. Conversely, essential oils from European *Bupleurum* species are characterized by elevated levels of *α*- and *β*-pinene, limonene, and 1,8-cineole, which might be attributed to environmental factors or genetic variations [1,10].

Despite potential differences in distribution and morphology, two annual representatives belonging to the Flora of Serbia [13], *Bupleurum praealtum* L. and *Bupleurum affine* L., share the same taxonomic subsection (Juncea) [14]. The essential oil of *B. praealtum* has only been investigated once previously, and a total of 86.9% of the constituents were identified, with the most abundant ones being (+)-spathulenol (17.7%), (–)-(*E*)-caryophyllene oxide (6.1%), octyl 2-methylbutanoate (5.8%), and 6,10,14-trimethylpentadecan-2-one (5.1%) [15]. In the diethyl ether extract of the aerial parts of this taxon, a series of new esters of stereoisomeric tetradeca-5,7,9,11-tetraen-1-ols, along with a tetra-unsaturated *γ*-tetradecalactone and dibenzylbutyrolactone lignan, was found [16]. Besides the flavonoid profile of *B. affine* [17], its essential oil has not been investigated up to date.

In this study, our objective is to enhance our understanding of the phytochemical diversity within the genus *Bupleurum*, which holds significant potential for both botanical classification and future pharmacological research. Utilizing comprehensive GC-MS analysis, we will investigate the chemical composition of essential oils extracted from *B. praealtum* schizocarps and, for the first time, *B. affine* aerial parts. Our primary focus will be on identifying and characterizing novel compounds, including conducting full NMR assignments. To verify the identity of selected constituents, we plan to perform appropriate synthesis and utilize the resulting standards for validating tentative identifications through co-injection experiments.

## 2. Results and Discussion

GC-MS analysis of the essential oils of *B. affine* (BA) and *B. praealtum* (BP) led to the identification of 230 constituents (Table 1), amounting to 97.1% and 91.1% of the total detected GC-peak areas, respectively. The oil isolated from BA aerial parts exhibits only slightly lower overall percentages of sesquiterpene hydrocarbons (41.2%) compared to the schizocarps oil of BP (45.3%), indicating a similarity in the predominance of sesquiterpene hydrocarbons in both oils. Additionally, BP oil contains a higher percentage of structurally and biochemically distinct constituents (“others”, 30.0%) compared to BA (12.7%), originating from a more diverse array of minor constituents. However, BP oil demonstrates a notably lower proportion of alkanes (5%) compared to BA (23.4%), implying potential differences in volatility and scent characteristics. The BP schizocarps essential oil predominantly consisted of germacrene D (24.0%), (*E*)-phytol (14.2%), and bicyclogermacrene (11.4%). Conversely, the primary constituents of the BA oil included undecane (21.0%), absent in the BP oil, along with germacrene D (18.6%) and (*E*)-phytol (5.0%).

**Figure 1:**
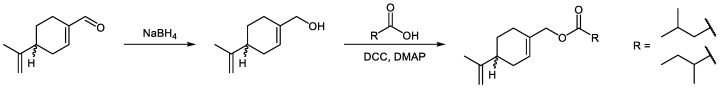
Synthesis of perillyl 2-methylbutanoate and perillyl 3-methylbutanoate.

Additionally, the GC-MS analysis of the BA essential oil revealed the presence of one minor constituent (RI 1664), with an MS fragmentation pattern indicating a perillyl ester, and a molecular ion at *m*/*z* 236 (Supplementary Materials Figure S1), assumed to be the ester of perilla alcohol and a five-carbon atom acid. Previously, these esters were identified only once in the essential oil of another Apiaceae species, *Kitagawia baicalensis* (Redowsky ex Willd.) Pimenov [20]. However, the paper did not specify the method used to confirm the identities of perillyl 2-methylbutanoate and perillyl 3-methylbutanoate. Solely comparing the retention indices provided (RI 1658 for perillyl 2-methylbutanoate and 1665 for perillyl 3-methylbutanoate) with the retention index of the unidentified component in the BA oil (RI 1664) does not definitively determine which of these two esters is present. Therefore, we opted to synthesize them for clarification. A reduction of the commercially available perilla aldehyde, followed by esterification with an appropriate acid gave the desired target esters (Figure 1). A co-injection experiment confirmed the occurrence of perillyl 2-methylbutanoate in the BA oil. The retention indices obtained from our synthesized standards do not align with those reported in the literature [20]. This discrepancy suggests a potential confusion in the identity of these esters by Letchamo et al. [20], as our data indicate that Letchamo’s 3-methylbutanoate closely matches our synthesized 2-methylbutanoate index. Consequently, we propose a reconsideration of the esters’ identities. Our study represents the first definitive confirmation of the natural occurrence of 2-methylbutanoate in this context. The absence of perilla alcohol and perilla aldehyde in the essential oil is intriguing, as it is closely biosynthetically related to perillyl esters. Most likely, perillyl derivatives are derived from an enzymatic allylic oxidation of limonene present in the BA oil (1.0%).

**Figure 2:**
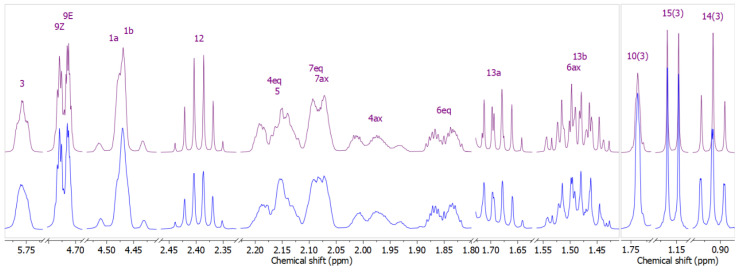
Upper trace: simulated ^1^H NMR (400 MHz) spectrum of perillyl 2-methylbutanoate; lower trace: ^1^H NMR (400 MHz, CDCl_3_) spectrum of perillyl 2-methylbutanoate (diastereomer mixture).

As there are two chiral centers in perillyl 2-methylbutanoate two diastereomers are possible. The synthetic sample was comprised of their unresolvable mixture on the DB-5MS column, while the NMR signals of these two diastereomers were also practically indistinct as will be described below. The spectra of the mixture of the synthesized esters were assigned with the aid of ^1^H NMR manual full spin spectral simulation (Figure 2, Table 2). The full spin analysis was performed by manually adjusting *δ*_H_ and *J* values to fit the experimentally available values and further optimized using MestReNova 11.0.3 software (tools/spin simulation). Although the recorded spectra represent the superimposed spectra of diastereomers (Supplementary Materials Figures S3 and S4), while the simulated spectra come from one diastereomer, the simulation outcome was in excellent agreement with the experimental data of the synthetic compound. This can be explained by the fact that the chiral centers are distant from one another within the molecule, resulting in no significant differences in the position and appearance of signals. These differences (mostly barely observable broadening) are visible only in certain signals, in the proximity of chiral centers (e.g., methyl group near the chiral center of the acidic part of the ester).

**Figure 3:**
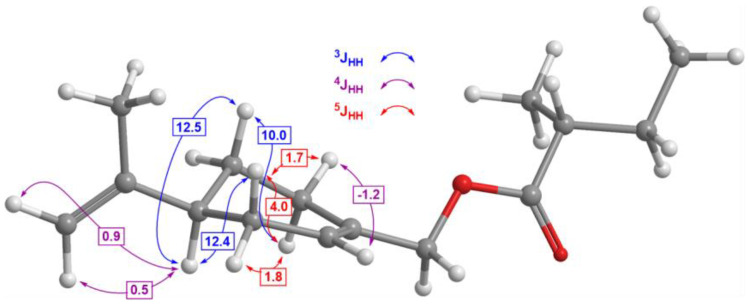
Three-dimensional structure of perillyl 2-methylbutanoate and the analysis of coupling constants disclosed using spin simulation.

Spectral simulation (Figure 2) allowed us to clearly discern the major couplings present among protons standardly buried within signals of higher order. The most significant coupling constants are shown in the structure in Figure 3. Three large constants, greater than 10 Hz, confirmed the approximately antiperiplanar position of hydrogens on the six-membered ring, placing the isopropylene group in a pseudo-equatorial position, as expected. Additionally, we noticed a large homoallylic constant of 4 Hz between the axial hydrogens in positions 4 and 7, besides two other homoallylic constants, of around 2 Hz. The reason for such a strong interaction between relatively distant hydrogen atoms can only be sought from their relative positions to the double bond, the parallel orientation of σ_C-H_ and π_C=C_, which further confirms the depicted 3D structure (Figure 3). The large value of one more long-range constant, the W-coupling constant of around 2 Hz, between equatorial hydrogens in positions 4 and 6, also confirmed the reliability of the depicted 3D structure of perillyl ester.

The four possible stereoisomers of perillyl 2-methylbutanoate could be expected to have different scents as well as potentially different biological activities. The synthesized mixture of these isomers (all four) had a floral-menthol scent. Synthesizing these esters using chirally pure alcohols and acids would allow us to determine the scent of each individual stereoisomer.

In the BA essential oil, the presence of numerous components with identical or similar mass spectra to esters of tetradec-4,6,8,10-tetraen-1-ol and acids with five carbon atoms, previously detected in the BP diethyl ether extract (praealtaesters A, B, C, and D), was noted. It is presumed that along the known esters, the remaining detected esters represent related constituents differing in the configuration of double bonds in the alcohol part of the molecule. It is interesting to note that such compounds were not detected in the BP essential oil. This discrepancy could be attributed to environmental factors or the fact that the essential oil was derived from the fruits of this plant species, while these polyunsaturated compounds were identified in the diethyl ether extract of the whole aerial parts. All the detected isomers would represent new natural products.

Furthermore, similar MS fragmentation patterns of two minor constituents of the BA oil (RI 1304 and 1394, and a base ion at *m*/*z* 43, which is indicative of acetates), and second-in-intensity ion at *m*/*z* 115 suggested that these constituents represent homologous acetates of long-chain saturated 4-alkanols. The alternative *α*-fragmentation of the 4-alkyl acetates observed at *m*/*z* 157, i.e., *m*/*z* 171, in the two spectra, led to the possible number of carbon atoms in the chains to be 10 and 11, respectively. The presence of 4-decyl acetate and 4-undecyl acetate, new natural compounds, was confirmed using the correlation of experimental RI data with available data from the literature in the case of 4-nonyl acetate [21]. In addition, isomeric undecanols (differing in the position of the alcohol group) were detected in the BA essential oil, likely formed through the hydroxylation of undecane present in the oil.

Interestingly, the essential oils of *Hypericum* spp. (Hypericaceae) and *Scandix pecten-veneris* L. (Apiaceae) also showcase a significant presence of C_9_–C_15_ alkanes, mirroring the composition of BA oil. For example, the essential oils extracted from *Hypericum* species from Bulgaria predominantly featured 2-methyloctane, ranging from 9.13% to 40.9%, alongside nonane and undecane [22]. Similarly, the essential oils from different *Hypericum* species from Serbia unveiled substantial alkane content, with *H. hirsutum* exhibiting heightened levels of nonane and undecane [23]. The alkane fraction in the essential oil of *S. pecten-veneris* was particularly prominent in samples obtained from aerial parts and roots, constituting 47.8% to 78.1% of the oils [24]. Although these compounds were also present in the fruits, their relative abundance was significantly lower (11.1%). Notably, there was a remarkably high concentration of tridecane and pentadecane in the oils of this plant species. This composition aligns with the findings observed in BA oil, where undecane is identified as one of the principal components (21%), whereas BP lacks undecane and similar chain-length alkanes. It is notable that the previously analyzed essential oil from *B. praealtum* aerial parts contained significant compounds such as (+)-spathulenol, (–)-(*E*)-caryophyllene oxide, and octyl 2-methylbutanoate, which were either present in significantly lower quantities or absent in the schizocarp essential oil investigated in this study. The study by Kapetanos et al. [15] did not specify which parts of the plant constituted the aerial parts they utilized, but based on the collection date (June 2003) from natural populations, it can be inferred that during this period, the plants were not in the fruit-bearing phase and thus did not contain schizocarps. This difference in plant phase could also explain the observed disparity in chemical composition between the schizocarp oil analyzed in this study and the previously analyzed aerial parts oil.

All the essential oils isolated from the *Bupleurum* species within the Juncea subsection, including *B. cappadocicum*, *B. gerardii*, and *B. pauciradiatum*, were characterized by a high content of undecane [10]. However, also significant differences were noted among these oils. For instance, in *B. cappadocicum*, the flower oil additionally contained high levels of heptanal, whereas the fruit oil was rich in spathulenol, and the root oil featured hexadecanoic acid [25]. In contrast, *B. gerardii* oils showed varying levels of hexanal across different plant parts, with undecane consistently present in high amounts [25,26]. Similarly, in *B. pauciradiatum*, germacrene D dominated in flower oils, *β*-pinene in fruit oils, and spathulenol in root oils, highlighting distinct chemical profiles influenced by plant organ specificity within the same subsection [27]. These findings underscore the variability in chemical profiles among *Bupleurum* species within the Juncea subsection, influenced by both genetic factors and environmental conditions. The two species analyzed in this study exhibit chemical traits similar to those observed in previously investigated oils from taxa within this subsection. It seems that there may be speciation within these species concerning the accumulation or biosynthesis of volatile alkanes or sesquiterpenes, which are major constituents of the oils. This warrants further investigation and could potentially yield chemotaxonomically significant traits.

## 3. Materials and Methods

### 3.1. Plant Material

The above-ground plant parts of *B. affine* in the intermediate flowering-fruit-bearing phase were collected in September 2016 on the slopes of Suva Planina Mt. (near Niš, southeastern Serbia, 43°11′53.1″ N 22°08′33.6″ E), and the schizocarps of *B. praealtum* were collected in September 2023 in the village Sićevo (southeastern Serbia), both from single populations. Voucher specimens have been deposited in the Herbarium of the Faculty of Sciences and Mathematics, University of Niš (voucher nos. HMN 12112 and HMN 18286). The plant material was identified by the late Professor Vladimir Ranđelović.

### 3.2. Isolation of Essential Oils

Dried above-ground parts of *B. affine* (120 g) and schizocarps of *B. praealtum* (100 g) were subjected to hydrodistillation for 2.5 h using the original Clevenger-type apparatus, and yielded 0.06% (*w*/*w*) and 0.01% (*w*/*w*) of essential oil, respectively. The distillation procedure was conducted in triplicate. The oils were taken in 2 mL of GC-grade pentane, dried with anhydrous Na_2_SO_4_, and immediately analyzed.

### 3.3. General Experimental Procedures

All used chemicals and solvents were obtained from commercial sources (Sigma-Aldrich, St. Louis, MO, USA; Merck, Darmstadt, Germany; Fisher Scientific, Waltham, MA, USA) and used as received, except for the solvents, which were predistilled and dried before use. Silica gel 60, particle size distribution 40–63 mm (Acros Organics, Geel, Belgium), was used for dry-flash chromatography, whereas precoated Al silica gel plates (Merck, Darmstadt, Germany), Kieselgel 60 F_254_, 0.2 mm) were used for analytical TLC analyses. The spots on TLC were visualized by spraying with 50% (*v*/*v*) aq. H_2_SO_4_ followed by heating. Elemental analysis (microanalysis of carbon and hydrogen) was carried out with a Carlo Erba Elemental Analyzer model 1106 (Carlo Erba Strumentazione, Milan, Italy). ^1^H and ^13^C NMR spectra were recorded on a Bruker Avance III 400 MHz NMR spectrometer (Fällanden, Switzerland; ^1^H at 400 MHz, ^13^C at 100.6 MHz), equipped with a 5 mm dual ^13^C/^1^H probe head at 20 °C. All the NMR spectra were recorded in chloroform-*d* (Sigma-Aldrich, St. Louis, MO, USA) with tetramethylsilane as the internal standard. Chemical shifts (*δ*) are reported in ppm and referenced to tetramethylsilane (*δ*_H_ = 0.00 ppm), or the (residual) solvent signal (CHCl_3_), and ^13^CDCl_3_, in ^1^H NMR and ^13^C NMR and heteronuclear 2D spectra, respectively. Scalar couplings are reported in Hertz (Hz). The acquired NMR experiments, both 1D and 2D, were recorded using standard Bruker built-in pulse sequences. ^1^H NMR full spin analysis of perillyl 2-methylbutanoate was performed by manually adjusting *δ*_H_ and *J* values to fit the experimentally available values and further optimized using MestReNova 11.0.3 software (tools/spin simulation). This procedure led to a systematic refinement of all calculated NMR parameters until the simulation outcome was in excellent agreement (NRMSD < 0.05%) with the experimental data of the isolated compounds.

### 3.4. Gas Chromatography–Mass Spectrometry (GC-MS) Analyses

GC-MS analyses (3 repetitions) were carried out using a Hewlett-Packard 6890N gas chromatograph equipped with a fused silica capillary column DB-5MS (5% diphenylsiloxane and 95% dimethylsiloxane, 30 m × 0.25 mm, film thickness 0.25 μm, Agilent Technologies, Palo Alto, CA, USA) and coupled with a 5975B mass selective detector from the same company. The injector and interface were operated at 250 and 300 °C, respectively. Oven temperature was raised from 70 to 290 °C at a heating rate of 5 °C/min and the program ended with an isothermal period of 10 min. As a carrier gas helium at 1.0 mL/min was used. The samples, 1.0 μL of essential oil solutions in diethyl ether (1.0 mg of an essential oil sample per 1.0 mL of solvent), were injected in a pulsed split mode (the flow was 1.5 mL/min for the first 0.5 min and then set to 1.0 mL/min throughout the remainder of the analysis; split ratio 40:1). MS conditions were as follows: ionization voltage 70 eV, acquisition mass range *m*/*z* 35–650, scan time 0.32 s. Constituents were identified by comparison of their linear retention indices (relative to C_8_–C_40_ *n*-alkanes on a DB-5MS column) with literature values and their mass spectra with those of authentic standards, as well as those from Wiley 6, NIST11, MassFinder 2.3, and a homemade MS library, except in the cases of 4-nonyl acetate [21] and dodecyl benzoate [28], with the spectra corresponding to pure substances and components of known oils, and wherever possible, by co-injection with an authentic sample.

### 3.5. Gas Chromatography–Flame Ionization Detector (GC-FID) Analyses

The GC-FID analyses (three repetitions of each sample) were carried out using an Agilent 7890A GC system equipped with a single injector, one flame ionization detector (FID), and a fused silica capillary column HP-5MS (5% diphenylsiloxane and 95% dimethylsiloxane, 30 m × 0.32 mm, film thickness 0.25 μm, Agilent Technologies, Palo Alto, CA, USA). The oven temperature was programmed from 70 °C to 300 °C at 15 °C/min and then held isothermally at 300 °C for 5 min; carrier gas was nitrogen at 3.0 mL/min; the injector temperature was held at 250 °C. The samples, 1.0 μL of the corresponding solutions, were injected in a splitless mode. The parameters of the FID detector were as follows: heater temperature—300 °C, H_2_ flow—30 mL/min, air flow—400 mL/min, makeup flow—23.5 mL/min, data collection—Agilent GC Chemstation with a digitization rate of 20 Hz.

### 3.6. Synthesis of Perilla Alcohol

A mixture of perilla aldehyde (450 mg, 3 mmol) and NaBH_4_ (456 mg, 12 mmol) in anhydrous methanol (25 mL) was stirred at 0 °C for one hour, then the ice bath was removed, and the stirring was continued for one hour at room temperature. The reaction mixture was quenched by slowly adding 1 M HCl until the excess borohydride was destroyed. The mixture was extracted with Et_2_O (3 × 50 mL). The organic layers were combined, washed with brine, dried with anhydrous MgSO_4_ and the solvent was removed in vacuo, giving 387 mg of perilla alcohol (yield 85%). Mass spectrum and RI of the synthesized alcohol ((4-(prop-1-en-2-yl)cyclohex-1-en-1-yl)methanol) matched with the data available in the literature [18].

### 3.7. Synthesis of Perillyl 2-Methylbutanoate and Perillyl 3-Methylbutanoate

A solution of perilla alcohol (152 mg, 1 mmol), 2-methylbutanoic acid (102 mg, 1 mmol), 4-(dimethylamino)pyridine (DMAP, 24 mg, 0.2 mmol), and *N*,*N*′-dicyclohexylcarbodiimide (DCC, 206 mg, 1 mmol) in 10 mL of dry CH_2_Cl_2_ was stirred in a round bottom flask overnight at room temperature, under argon. Afterward, the solvent was removed in vacuo; then, 10 mL of cold pentane was added to the residue, and the precipitated *N*,*N*′-dicyclohexylurea was filtered off. The filtrate was concentrated in vacuo, and the resulting residue was purified by silica gel column chromatography giving 177 mg (75% yield) of perillyl 2-methylbutanoate.

A solution of perilla alcohol (15.2 mg, 0.1 mmol), 3-methylbutanoic acid (10.2 mg, 0.1 mmol), 4-(dimethylamino)pyridine (DMAP, 2.4 mg, 0.02 mmol), and *N*,*N*′-dicyclohexylcarbodiimide (DCC, 20.6 mg, 0.1 mmol) in 1 mL of dry CH_2_Cl_2_ was stirred in a round bottom flask overnight at room temperature in a GC vial. Afterward, the reaction mixture was filtered through a thin layer of Celite^®^, and the resulting residue was analyzed by GC-MS, without isolation, to obtain the MS and RI data. The resulting reaction mixture was purified by silica gel column chromatography giving 19.5 mg (83% yield) of perillyl 3-methylbutanoate and was used to obtain NMR data.

*Perillyl 2-methylbutanoate-(4-(prop-1-en-2-yl)cyclohex-1-en-1-yl)methyl 2-methylbutanoate.* Colorless liquid; RI (DB-5MS) 1664. MS (EI), (*m*/*z*, (relative abundance, %)): 236 (2), 134 (62), 119 (100), 106 (50), 105 (42), 93 (56), 92 (66), 91 (85), 79 (40), 57 (92), 41 (36). ^1^H-NMR (400 MHz, CDCl_3_) and ^13^C-NMR (100.6 MHz, CDCl_3_) are given in Table 2. Elemental analysis found: C 76.25; H 10.22; O 13.53; Calcd. C 76.23; H 10.23; O 13.54.

*Perillyl 3-methylbutanoate-(4-(prop-1-en-2-yl)cyclohex-1-en-1-yl)methyl 3-methylbutanoate.* Colorless liquid; RI (DB-5MS) 1672. MS (EI), (*m*/*z*, (relative abundance, %)): 236 (2), 134 (64), 119 (100), 106 (49), 105 (43), 93 (55), 92 (66), 91 (86), 85 (56), 79 (38), 57 (60). ^1^H NMR (CDCl_3_) *δ* 0.96 (d, *J* = 6.6 Hz, 6 H, CH_3_-14, and CH_3_-15), 1.43–1.55 (m, 1 H, CH-6b), 1.74 (m, 3 H, CH_3_-10), 1.81–1.89 (m, 1 H, CH-6a), 1.91–2.03 (m, 1 H, CH-4b), 2.05–2.20 (overlapped multiplets, 5 H, CH-5, CH-4a, CH_2_-7, CH-13), 2.19–2.23 (m, 2 H, CH_2_-12), 4.43–4.50 (m, 2 H, CH_2_-1), 4.71 (m, 1 H, CH-9E), 4.73 (quint, *J* = 1.5 Hz, 1 H, CH-9Z), 5.76 (m, 1 H, CH-3); ^13^C NMR (CDCl_3_) *δ* 20.89 (C-10), 22.57 (C-14 and C-15), 25.88 (C-13), 26.56 (C-7), 27.46 (C-6), 30.60 (C-4), 40.97 (C-5), 43.64 (C-12), 68.34 (C-1), 108.91 (C-9), 125.86 (C-3), 132.87 (C-2), 149.76 (C-8), 173.24 (C-11).

## 4. Conclusions

In conclusion, our study presents a detailed characterization of the essential oils extracted from *B. praealtum* (BP) and *B. affine* (BA), revealing their distinct chemical compositions through comprehensive GC-MS analysis. We identified a total of 230 constituents across both oils. In BP schizocarps oil, major components included germacrene D (24.0%), (*E*)-phytol (14.2%), and bicyclogermacrene (11.4%). In contrast, BA oil was characterized by significant levels of undecane (21.0%), absent in BP, along with germacrene D (18.6%) and (*E*)-phytol (5.0%). Notably, we confirmed the presence of perillyl 2-methylbutanoate in BA oil for the first time using a synthetic approach, employing advanced spectroscopic techniques to characterize its structure. The identification of isomeric praealtaesters in BA oil underscores its chemical complexity. Additionally, the detection of homologous acetates like 4-decyl acetate and 4-undecyl acetate in BA oil expands the known chemical diversity within the *Bupleurum* genus. These findings suggest potential ecological adaptations or variations in biosynthetic pathways among *Bupleurum* species. Overall, our study enhances the understanding of these plants’ phytochemical profiles and their ecological significance.

## Figures and Tables

**Table 1:** Chemical composition of *B. affine* and *B. praealtum* essential oils.

RI ^1^	RI ^2^	Compound ^3^	Content ^4^		Class ^5^
			BA	BP	
770	768	(*Z*)-2-Penten-1-ol	tr ^6^	- ^7^	GL
802	801	Hexanal ^8^	tr	-	GL
830	828	Furfural ^8^	tr	-	O
845	844	(*E*)-3-Hexenol ^8^	tr	-	GL
853	850	(*Z*)-3-Hexenol ^8^	2.3	-	GL
862	859	(*Z*)-2-Hexenal ^8^	tr	-	GL
868	863	1-Hexanol ^8^	0.7	-	GL
900	900	Nonane ^8^	0.1	-	A
904	901	Heptanal ^8^	0.1	-	GL
927	924	*α*-Thujene	tr	-	MH
936	932	*α*-Pinene ^8^	0.2	-	MH
953	946	Camphene ^8^	tr	-	MH
959	947	(*E*)-2-Heptenal ^8^	tr	-	GL
968	959	1-Heptanol ^8^	tr	-	GL
972	952	Benzaldehyde ^8^	tr	tr	O
975	969	Sabinene	0.4	-	MH
978	974	1-Octen-3-ol ^8^	tr	-	GL
983	974	*β*-Pinene ^8^	0.1	-	MH
985	981	6-Methyl-5-hepten-2-one ^8^	tr	-	O
989	984	2-Pentylfuran	tr	-	GL
989	988	Myrcene ^8^	0.7	-	MH
1000	1000	Decane ^8^	tr	-	A
1004	1004	(*Z*)-3-Hexenyl acetate ^8^	0.6	-	GL
1005	998	Octanal ^8^	tr	-	GL
1010	1007	Hexyl acetate ^8^	0.1	-	GL
1018	1014	(2*E*,4*E*)-2,4-Nonadiene	-	tr	O
1020	1005	(2*E*,4*E*)-2,4-Heptadienal	tr	-	GL
1020	1014	*α*-Terpinene ^8^	tr	-	MH
1024	/	6,6-Dimethyl-2-cyclohexenone ^9^	tr	-	O
1027	1020	*p*-Cymene ^8^	0.1	-	MH
1032	1024	Limonene ^8^	1.0	-	MH
1034	1025	*β*-Phellandrene ^8^	tr	-	MH
1038	1033	2,2,6-Trimethylcyclohexanone	tr	-	O
1044	1044	*β*-Isophorone (syn. 3,5,5-trimethyl-3-cyclohexen-1-one)	tr	-	O
1045	1044	(*E*)-*β*-Ocimene	tr	-	MH
1049	1036	Phenylacetaldehyde ^8^	0.1	0.2	O
1060	1054	*γ*-Terpinene ^8^	tr	-	MH
1061	1049	(*E*)-2-Octenal	tr	-	GL
1063	1057	2,6,6-Trimethyl-2-cyclohexenone ^8^	tr	-	O
1070	1060	(*E*)-2-Octen-1-ol	tr	-	GL
1070	1063	1-Octanol ^8^	0.1	tr	GL
1074	1065	*cis*-Sabinene hydrate	tr	-	MO
1090	1086	Terpinolene ^8^	tr	-	MH
1092	1098	1-Undecene	0.2	-	O
1100	1100	Undecane ^8^	21.0	-	A
1101	1100	Nonanal ^8^	-	tr	GL
1125	1108	1,3,8-*p*-Menthatriene	tr	-	MH
1125	1127	2,6,6-Trimethyl-2-cyclohexene-1-carboxaldehyde (syn. *α*-Cyclocitral) ^8^	tr	-	O
1163	1157	(*E*)-2-Nonenal	0.1	tr	GL
1173	1165	1-Nonanol ^8^	tr	-	GL
1185	1174	Terpinen-4-ol ^8^	0.1	tr	MO
1200	1186	*α*-Terpineol ^8^	tr	-	MO
1200	1200	Dodecane ^8^	tr	tr	A
1205	1196	Safranal ^8^	tr	-	O
1208	1201	Decanal ^8^	0.1	tr	GL
1224	1217	*β*-Cyclocitral	0.1	-	O
1232	1232	*O*-Methyl thymol ^8^	0.1	-	MO
1235	1232	(*Z*)-3-Hexenyl 3-methylbutanoate ^8^	tr	-	GL
1241	1241	Hexyl isovalerate ^8^	tr	-	MO
1259	1261	(2,6,6-Trimethyl-1-cyclohexen-1-yl)acetaldehyde	tr	tr	O
1260	1262	(*E*)-2-Decenal	0.1	0.2	GL
1274	1266	1-Decanol ^8^	tr	-	GL
1289	1288	3-Undecanone ^8^	tr	-	O
1292	1293	Dihydroedulan I	0.1	tr	O
1292	1286	4-Undecanol	tr	-	O
1294	1294	2-Undecanone ^8^	tr	-	O
1297	1298	1-Tridecene	tr	-	O
1297	1289	Thymol ^8^	tr	-	MO
1300	1300	Tridecane ^8^	0.4	-	A
1300	1293	3-Undecanol	tr	-	O
1302	1299	Theaspirane (isomer 1)	tr	tr	O
1304	1301	2-Undecanol	0.1	-	O
1304	/	4-Decyl acetate ^10^	tr	-	
1309	1305	Undecanal ^8^	tr	0.1	GL
1318	1313	Theaspirane (isomer 2)	tr	tr	O
1320	1309	*p*-Vinylguaiacol ^8^	tr	-	O
1324	1315	(*E*,*E*)-2,4-Decadienal	0.1	0.1	GL
1336	1335	*δ*-Elemene	tr	2.0	SH
1347	1345	7-*epi*-Silphiperfol-5-ene	tr	-	SH
1349	1345	*α*-Cubebene	tr	tr	SH
1358	1355	Dehydro-*ar*-ionene	tr	tr	O
1361	1361	(*Z*)-*β*-Damascenone	tr	-	O
1368	1365	(*Z*)-2-Undecenal	0.1	0.1	GL
1372	1374	Cycloisosativene	0.1	-	SH
1373	1373	*α*-Ylangene	tr	-	SH
1379	1374	*α*-Copaene	0.2	0.2	SH
1382	1383	(*E*)-*β*-Damascenone	0.2	tr	O
1387	1387	*β*-Bourbonene	0.3	2.2	SH
1390	1387	*β*-Cubebene	tr	-	SH
1392	1389	*β*-Elemene	0.9	0.7	SH
1394	/	4-Undecyl acetate ^10^	tr	-	O
1400	1400	Tetradecane ^8^	tr	tr	A
1404	1405	Sesquithujene	0.3	-	SH
1407	1412	6,10-Dimethyl-2-undecanone	-	tr	O
1411	1407	1-Decyl acetate ^8^	1.4	-	GL
1417	1412	Dodecanal ^8^	-	0.4	GL
1424	1417	(*E*)-Caryophyllene ^8^	4	-	SH
1424	1419	*β*-Ylangene	-	2.2	SH
1430	1437	*α*-Guaiene	0.2	-	SH
1436	1431	*β*-Gurjunene	2.5	-	SH
1437	1430	*β*-Copaene	-	1.0	SH
1442	1446	Sesquisabinene	0.2	-	SH
1446	1439	Isogermacrene D	-	0.5	SH
1453	1458	*allo*-Aromadendrene	tr	-	SH
1455	1453	Geranyl acetone	-	1.6	O
1456	1454	(*E*)-*β*-Farnesene ^8^	4.7		SH
1460	1452	*α*-Humulene ^8^	1.7	tr	SH
1477	1469	1-Dodecanol ^8^	-	0.3	GL
1493	1484	Germacrene D ^8^	18.6	24.0	SH
1496	1489	*β*-Selinene	0.2	-	SH
1498	1493	*α*-Zingiberene	0.3	-	SH
1498	1500	*α*-Muurolene	tr	-	SH
1501	1500	Bicyclogermacrene	2.8	11.4	SH
1500	1500	Pentadecane ^8^	tr	-	A
1506	1505	(*E*,*E*)-*α*-Farnesene ^8^	tr	-	SH
1510	1503	*β*-Dihydroagarofuran	tr	-	SO
1513	1508	Germacrene A	1.3	-	SH
1513	1514	*β*-Curcumene	tr	-	SH
1514	1504	Cuparene	tr	-	SH
1518	1513	*γ*-Cadinene	0.4	-	SH
1520	1514	Cubebol	tr	-	SO
1524	1522	*δ*-Cadinene	2.1	1.1	SH
1525	1521	*β*-Sesquiphellandrene	tr	-	SH
1526	1528	Zonarene	0.2	-	SH
1529	1529	(*E*)-*γ*-Bisabolene	tr	-	SH
1535	1529	Kessane	0.5	-	SO
1535	1528	*cis*-Calamenene	tr	-	SH
1535	1533	10-*epi*-Cubebol	tr	-	SO
1536	1533	*trans*-Cadina-1,4-diene	tr	-	SH
1541	1537	*α*-Cadinene	0.2	tr	SH
1541	1534	Liguloxide	tr	-	SO
1545	1544	*α*-Calacorene	tr	tr	SH
1552	1548	*α*-Agarofuran	0.1	-	SO
1552	1550	*cis*-Muurol-5-en-4*β*-ol	tr	-	SO
1562	1561	(*E*)-Nerolidol	0.1	0.1	SO
1572	1565	Dodecanoic acid ^8^	0.1	-	O
1575	1577	(*E*)-Dendrolasin	-	tr	SO
1576	1565	(*Z*)-3-Hexenyl benzoate ^8^	tr	-	GL
1577	1567	(*E*)-2-Tridecenal	-	0.2	GL
1579	1574	Germacrene D-4-ol	tr	-	SO
1582	1577	Spathulenol ^8^	1.3	5.0	SO
1587	1581	10-*epi*-Junenol	0.6	-	SO
1595	1589	*Allo*-hedycaryol	1.9	-	SO
1598	1594	Salvial-4(14)-en-1-one	0.4	0.6	SO
1600	1600	Hexadecane ^8^	tr	tr	A
1609	1607	*β*-Oplopenone	0.5	-	SO
1610	1600	Rosifoliol	-	tr	SO
1607	1611	1-Dodecyl acetate ^8^	tr	0.5	GL
1615	1608	Humulene epoxide II	0.1	-	SO
1619	1618	1,10-di-*epi*-Cubenol	tr	-	SO
1626	1618	Junenol	-	tr	SO
1633	1627	1-*epi*-Cubenol	1.5	-	SO
1635	1631	(*E*)-Sesquilavandulol	-	0.5	SO
1639	1625	Isospathulenol	-	1.2	SO
1640	1641	*β*-Eudesmol	0.2	-	SO
1646	1640	*epi*-*α*-Murrolol (syn. *τ*-muurolol)	0.5	-	SO
1652	1645	*α*-Muurolol (syn. torreyol)	1.1	-	SO
1661	1652	*α*-Cadinol	1.9	0.1	SO
1664	1664	Perillyl 2-methylbutanoate ^8,11^	0.3	-	MO
1671	1666	Bulnesol	tr	-	SO
1671	1671	1-Tetradecanol ^8^	-	0.5	O
1677	1673	3-Methylhexadecane	0.6		A
1690	1685	Germacra-4(15),5,10(14)-trien-1*α*-ol	0.2	0.6	SO
1700	1698	2-Pentadecanone	0.2	-	O
1700	1700	Heptadecane ^8^	tr	-	A
1716	1715	Pentadecanal ^8^	0.1	0.1	O
1734	1739	(*E*)-Sesquilavandulyl acetate	-	0.3	SO
1745	1740	Mint sulfide	0.1	0.5	SO
1775	1759	Benzyl benzoate ^8^	-	0.3	O
1769	1765	Tetradecanoic acid ^8^	tr	0.5	O
1793	1789	1-Octadecene	tr	-	O
1800	1800	Octadecane ^8^	tr	tr	A
1804	1803	14-Hydroxy-*δ*-cadinene	tr	-	SO
1819	1818	Hexadecanal ^8^	0.1	0.2	O
1837	1830	Neophytadiene (Isomer 1)	1.3	0.5	O
1843	1843	Hexahydrofarnesyl acetone	0.4	3.7	O
1851	1843	6,10,14-Trimethylpentadecan-2-ol ^11^	tr	-	O
1861	1849	Neophytadiene (Isomer 3)	0.1	0.1	O
1883	/	Neophytadiene (Isomer 2) ^9^	-	0.2	O
1884	1880	1-Hexadecanol ^8^	tr	-	O
1900	1900	Nonadecane ^8^	0.2	0.3	A
1911	1913	(*E*,*E*)-5,9-Farnesyl acetone	tr	-	O
1920	1920	Heptadecanal ^8^	0.1	0.6	O
1926	1921	Methyl hexadecanoate ^8^	tr	0.1	O
1947	1942	Isophytol	tr	0.1	O
1977	1959	Hexadecanoic acid ^8^	0.3	6.0	O
1994	1990	Ethyl palmitate ^8^	tr	-	O
2000	2000	Eicosane ^8^	tr	-	A
2024	2026	(*E*,*E*)-Geranyl linalool	tr	0.2	O
2043	2035	(*Z*)-Falcarinol	0.4	-	O
2088	2083	1-Octadecanol ^8^	0.1	-	O
2097	2092	Methyl *γ*-linolenate	tr	0.2	O
2100	2100	Heneicosane ^8^	0.4	0.4	A
2117	2114	(*E*)-Phytol ^8^	5.0	14.2	O
2192	2172	1-Nonadecanol ^8^	0.1	-	O
2195	2196	1-Docosene	tr	-	O
2200	2200	Docosane ^8^	tr	0.1	A
2203	2213 ^10^	Dodecyl benzoate ^8^	0.1	-	O
2227	2225	Eicosanal	-	0.1	O
2230	2227	(5*Z*,7*E*,9*E*,11*E*)-Tetradeca-5,7,9,11-tetraen-1-yl 3-methylbutanoate (Praealtaester B)	0.1	-	O
2238	/	(5,7,9,11)-Tetradeca-5,7,9,11-tetraen-1-yl 3-methylbutanoate (isomer 1) ^9^	0.1	-	O
2273	/	(5,7,9,11)-Tetradeca-5,7,9,11-tetraen-1-yl 3-methylbutanoate (isomer 2) ^9^	0.1	-	O
2297	/	(5,7,9,11)-Tetradeca-5,7,9,11-tetraen-1-yl-2-hydroxy-3-methylbutanoate (isomer) ^9^	tr	-	O
2300	2300	Tricosane ^8^	0.1	-	A
2323	2329	Heneicosanal	-	tr	O
2346	2342	*δ*-Hexadecalactone	tr	-	O
2364	2352	4,8,12,16-Tetramethylheptadecan-4-olide ^11^	-	0.1	O
2370	2365	(5*Z*,7*E*,9*E*,11*E*)-Tetradeca-5,7,9,11-tetraen-1-yl (*R*)-2-hydroxy 3-methylbutanoate (Praealtaester A)	1.5	-	O
2395	2395	1-Tetracosene	0.1	tr	O
2400	2400	Tetracosane ^8^	0.3	0.2	A
2414	/	(5,7,9,11)-Tetradeca-5,7,9,11-tetraen-1-yl-2-hydroxy 3-methylbutanoate (isomer) ^9^	1.5	-	O
2430	2430	Docosanal	tr	-	O
2500	2500	Pentacosane ^8^	0.1	1.2	A
2514	/	(5,*7,*9,11)-Tetradeca-5,7,9,11-tetraen-1-yl 2-acetoxy 3 methylbutanoate (isomer) ^9^	tr	-	O
2594	2595	1-Hexacosene	tr	tr	O
2600	2600	Hexacosane ^8^	tr	0.5	A
2635	2630	Tetracosanal	tr	-	O
2700	2700	Heptacosane ^8^	0.1	1.7	A
2740	2735	Pentacosanal	tr	-	O
2794	2795	1-Octacosene	tr	-	O
2800	2800	Octacosane ^8^	tr	0.2	A
2811	2814	(*E*,*E*,*E*,*E*)-Squalene	tr	0.1	O
2841	2840	Hexacosanal	0.1	0.1	O
2900	2900	Nonacosane ^8^	0.1	0.4	A
2940	2944	Heptacosanal	-	tr	O
3040	3042	Octacosanal	tr	0.5	O
3082	3090	10-Nonacosanone	tr	-	O
3100	3100	Hentriacontane ^8^	-	tr	A
3213	3235	Triacontanal	tr	-	O
Total identified			97.1	91.1	
Alkanes			23.4	5.0	
Green leaf volatiles			5.8	1.9	
Monoterpene hydrocarbons			2.5	tr	
Oxygenated monoterpenes			0.5	tr	
Sesquiterpene hydrocarbons			41.2	45.3	
Oxygenated sesquiterpenes			11.0	8.9	
Others			12.7	30.0	

^1^ Retention indices determined experimentally on a DB-5MS column relative to a series of C_7_-C_40_ *n*-alkanes. ^2^ Literature values of retention indices taken from Adams [18] or NIST [19] collection, if not stated otherwise. ^3^ Compound identified based on mass spectra and retention indices matching with literature data, if not stated otherwise. ^4^ Values are means of three individual analyses. ^5^ A, alkanes; MH, monoterpene hydrocarbons; MO, oxygenated monoterpenes; SH, sesquiterpene hydrocarbons; SO, oxygenated sesquiterpenes; O, others. ^6^ tr, trace amount (<0.05%). ^7^ -, not detected. ^8^ Constituent identity confirmed by co-injection of an authentic sample. ^9^ Tentative identification based solely on MS comparison. ^10^ see Section 3.3. ^11^ Correct stereochemistry is unknown.

**Table 2:** ^1^H (400 MHz) and ^13^C (100.6 MHz) NMR data of perillyl 2-methylbutanoate (CDCl_3_, NMR parameters are derived from manual iterative full spin analysis), along with the observed grHMBC and NOESY correlations.

Position	δ_H_ (m, *J* (Hz), Integral) ^1^	δ_C_ (ppm)	grHMBC ^2^	NOESY ^3^
1a	4.4873 (ddtdd, ^2^*J*_1a,1b_ = −14.1, ^5^*J*_1a,4ax_ = 2.0, ^4^*J*_1a,7ax_ = ^4^*J*_1a,7eq_ = 1.8, ^4^*J*_1a,3_ = 1.4, ^5^*J*_1a,4eq_ = 1.25, 1 H)	68.24	2, 3, 7, 11	3, 7ax, 7eq
1b	4.4592 (ddtt, ^2^*J*_1a,1b_ = −14.1, ^4^*J*_1b,3_ = −1.35, ^5^*J*_1b,4ax_ = ^5^*J*_1b,4eq_ = 1.3, ^4^*J*_1b,7ax_ = ^4^*J*_1b,7eq_ = 0.7, 1 H)			
2	/	132.92	/	/
3	5.7572 (dddddd, ^3^*J*_3,4ax_ = 4.4, ^3^*J*_3,4eq_ = 3.45, ^4^*J*_3,7ax_ = −1.6, ^4^*J*_1a,3_ = 1.4, ^4^*J*_1b,3_ = −1.35, ^4^*J*_3,7eq_ = −1.2, 1 H)	125.60	2, 7, 4	4ax, 4eq, 1a, 1b
4ax	1.9770 (ddddddd, ^2^*J*_4ax,4eq_ = −18.7, ^3^*J*_4ax,5_ = 12.4, ^3^*J*_3,4ax_ = 4.4, ^5^*J*_4ax,7ax_ = 4.0, ^5^*J*_1a,4ax_ = 2.0, ^5^*J*_4ax,7eq_ = 1.7, ^5^*J*_1b,4ax_ = 1.3, 1 H)	30.57	2, 3, 5	3
4eq	2.1642 (ddddtdd, ^2^*J*_4ax,4eq_ = −18.7, ^3^*J*_3,4eq_ = 3.45, ^3^*J*_4eq,5_ = 2.8, ^4^*J*_4eq,6eq_ = 2.0, ^5^*J*_4eq,7ax_ = ^5^*J*_4eq,7eq_ = 1.8, ^5^*J*_1b,4eq_ = 1.3, ^5^*J*_1a,4eq_ = 1.25, 1 H)			
5	2.1585 (ddddddd, ^3^*J*_5,6ax_ = 12.5, ^3^*J*_5,4ax_ = 12.4, ^3^*J*_5,6eq_ = 4.8, ^3^*J*_5,4eq_ = 2.8, ^4^*J*_5,9E_ = 0.85, ^4^*J*_5,10_ = 0.55, ^4^*J*_5,9Z_ = 0.5, 1 H)	40.96	8, 9, 6	6a, 6b
6ax	1.4930 (dddd, ^2^*J*_6ax,6eq_ = −13.3, ^3^*J*_5,6ax_ = 12.5, ^3^*J*_6ax,7ax_ = 10.0, ^3^*J*_6ax,7eq_ = 7.0, 1 H)	27.42	5, 7	5, 7a, 7b
6eq	1.8500 (ddddd, ^2^*J*_6ax,6eq_ = −13.3, ^3^*J*_6eq,7ax_ = 6.0, ^3^*J*_5,6eq_ = 4.8, ^4^*J*_4eq,6eq_ = 2.0, ^3^*J*_6eq,7ax_ = 0.6, 1 H)			
7ax	2.0770 (ddddtdd, ^2^*J*_7ax,7eq_ = −17.5, ^3^*J*_6ax,7ax_ = 10.0, ^3^*J*_6eq,7ax_ = 6.0, ^5^*J*_4ax,7ax_ = 4.0, ^4^*J*_1a,7ax_ = ^5^*J*_4eq,7ax_ = 1.8, ^4^*J*_3,7ax_ = −1.6, ^4^*J*_1b,7ax_ = 0.7, 1 H)	26.93	2, 3, 6	6aq, 6eq, 1a, 1b
7eq	2.0870 (ddtdddd, ^2^*J*_7ax,7eq_ = −17.5, ^3^*J*_6ax,7eq_ = 7.0, ^4^*J*_1a,7eq_ = ^5^*J*_4eq,7eq_ = 1.8, ^5^*J*_4ax,7eq_ = 1.7, ^4^*J*_3,7eq_ = −1.2, ^4^*J*_1b,7eq_ = 0.7, ^3^*J*_6eq,7eq_ = 0.6, 1 H)			
8	/	149.72	/	/
9E	4.7134 (dqd, ^2^*J*_9E,9Z_ = 1.85, ^4^*J*_9E,10_ = 1.1, ^4^*J*_5,9E_ = 0.85, 1 H)	108.88	8, 10, 5	5
9Z	4.7296 (dqd, ^2^*J*_9E,9Z_ = 1.85, ^4^*J*_9Z,10_ = 1.5, ^4^*J*_5,9Z_ = 0.5, 1 H)			
10	1.7393 (ddd, ^4^*J*_9Z,10_ = 1.5, ^4^*J*_9E,10_ = 1.1, ^4^*J*_5,10_ = 0.55, 3 H)	20.87	5, 8, 9	5
11	/	176.78	/	/
12	2.3944 (tq, ^3^*J*_12,13a_ = ^3^*J*_12,13b_ = 7.1, ^3^*J*_12,15_ = 7.05, 1 H)	41.28	11, 13, 15	13, 15
13a	1.6942 (dqd, ^2^*J*_13a,13b_ = −17.2, ^3^*J*_13a,14_ = 7.4, ^3^*J*_12,13a_ = 7.1, 1 H)	26.47	12, 14	12, 14
13b	1.4820 (dqd, ^2^*J*_13a,13b_ = −17.2, ^3^*J*_13b,14_ = 7.4, ^3^*J*_12,13b_ = 7.1, 1 H)			
14	0.9118 (q, ^4^*J*_13,14_ = 7.4, 3 H)	11.78	12, 13	13a, 13b
15	1.1557 (d, ^3^*J*_12,15_ = 7.5, 3 H)	16.78	11, 12	12

^1^ Coupling constant values were initially inferred from ^1^H homoselective decoupling NMR experiments and afterward refined through a manual iterative full spin analysis. For details, cf. Experimental part. ^2^ grHMBC correlations observed between the hydrogen in this row and the carbon in the listed position. ^3^ Cross-peaks observed in the NOESY spectrum.

## Data Availability

Data are contained within the article and Supplementary Materials.

## Supplementary Materials

The following supporting information can be downloaded at: https://www.mdpi.com/article/10.3390/plants13152076/s1, Figure S1. EI (70 eV) mass spectrum of perillyl 2-methylbutanoate; Figure S2. EI (70 eV) mass spectrum of perillyl 3-methylbutanoate; Figure S3. ^1^H NMR spectrum of perillyl 2-methylbutanoate (diastereomer mixture); Figure S4. ^13^C NMR spectrum of perillyl 2-methylbutanoate (diastereomer mixture); Figure S5. ^1^H NMR spectrum of perillyl 3-methylbutanoate; Figure S6. ^13^C NMR spectrum of perillyl 3-methylbutanoate.
