# Supplementary material for: Essential Oil Composition of Bupleurum praealtum and Bupleurum affine: New Natural Constituents
Source: Plants (Basel). 2024 Jul 26;13(15):2076. doi: 10.3390/plants13152076 (PMC11314282; doi:10.3390/plants13152076)
Supplement: Supplementary file 1 [file plants-13-02076-s001.zip › plants-3093070-supplementary.pdf]

Supplementary material

for

Essential Oil Composition of *Bupleurum praealtum* and *Bupleurum affine*: New Natural Constituents

Milica D. Nešić[1], Milan S. Nešić[1], Milan Ž. Dimitrijević[1] and Niko S. Radulović[1,*]

[1] Department of Chemistry, Faculty of Sciences and Mathematics, University of Niš, Višegradska 33, 18000 Niš, Serbia

* Correspondence: nikoradulovic@yahoo.com

Content:

Figure S1. EI (70 eV) mass spectrum of perillyl 2-methylbutanoate
Figure S2. EI (70 eV) mass spectrum of perillyl 3-methylbutanoate
Figure S3. ^{1}H NMR spectrum of perillyl 2-methylbutanoate (diastereomer mixture)
Figure S4. ^{13}C NMR spectrum of perillyl 2-methylbutanoate (diastereomer mixture)
Figure S5. ^{1}H NMR spectrum of perillyl 3-methylbutanoate
Figure S6. ^{13}C NMR spectrum of perillyl 3-methylbutanoate

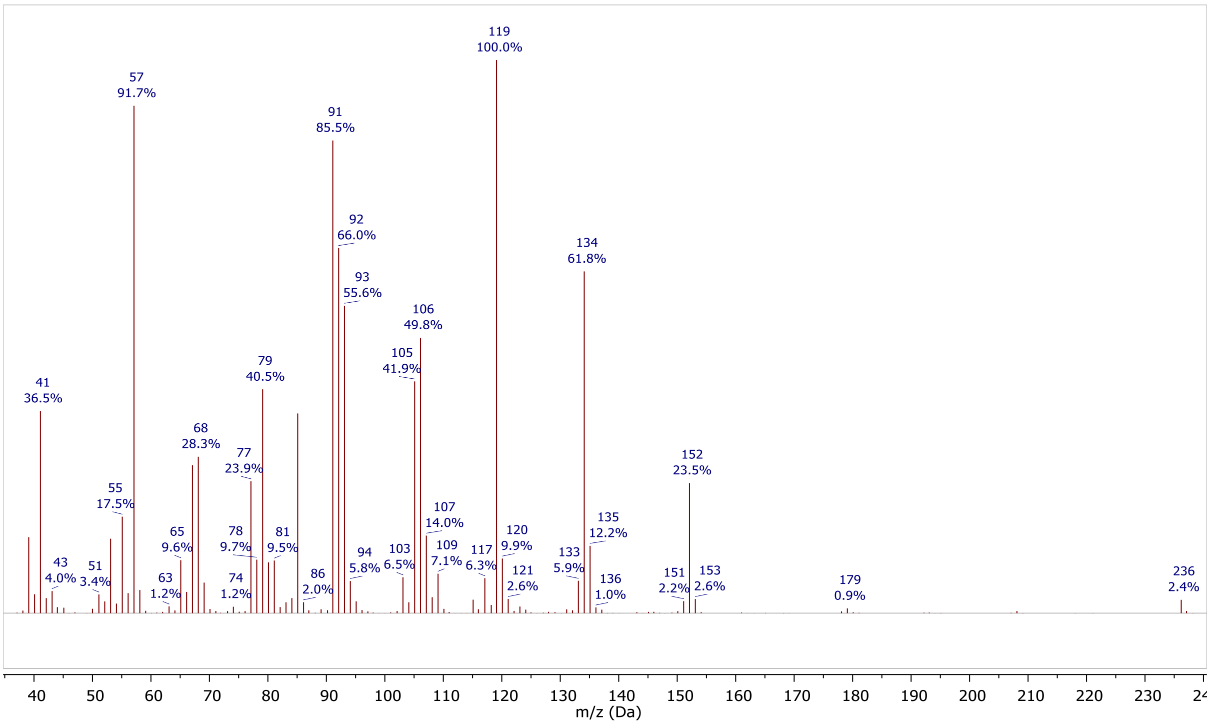

Figure S1. EI (70 eV) mass spectrum of perillyl 2-methylbutanoate

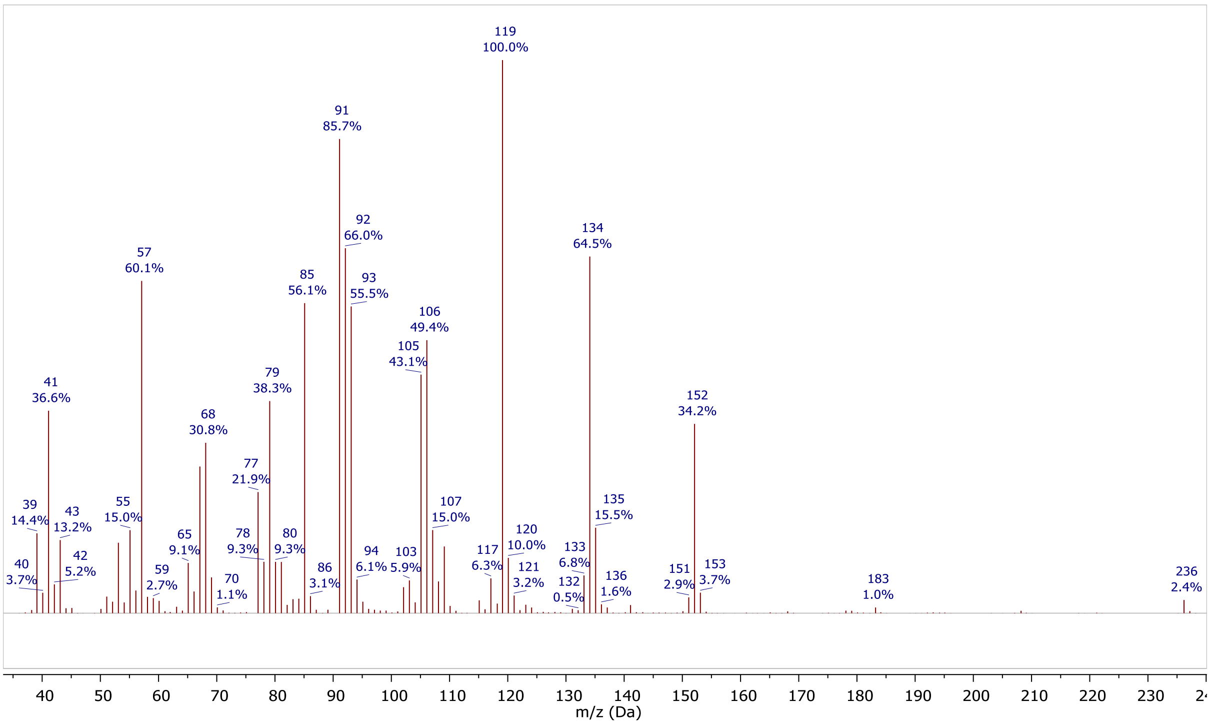

Figure S2. EI (70 eV) mass spectrum of perillyl 3-methylbutanoate

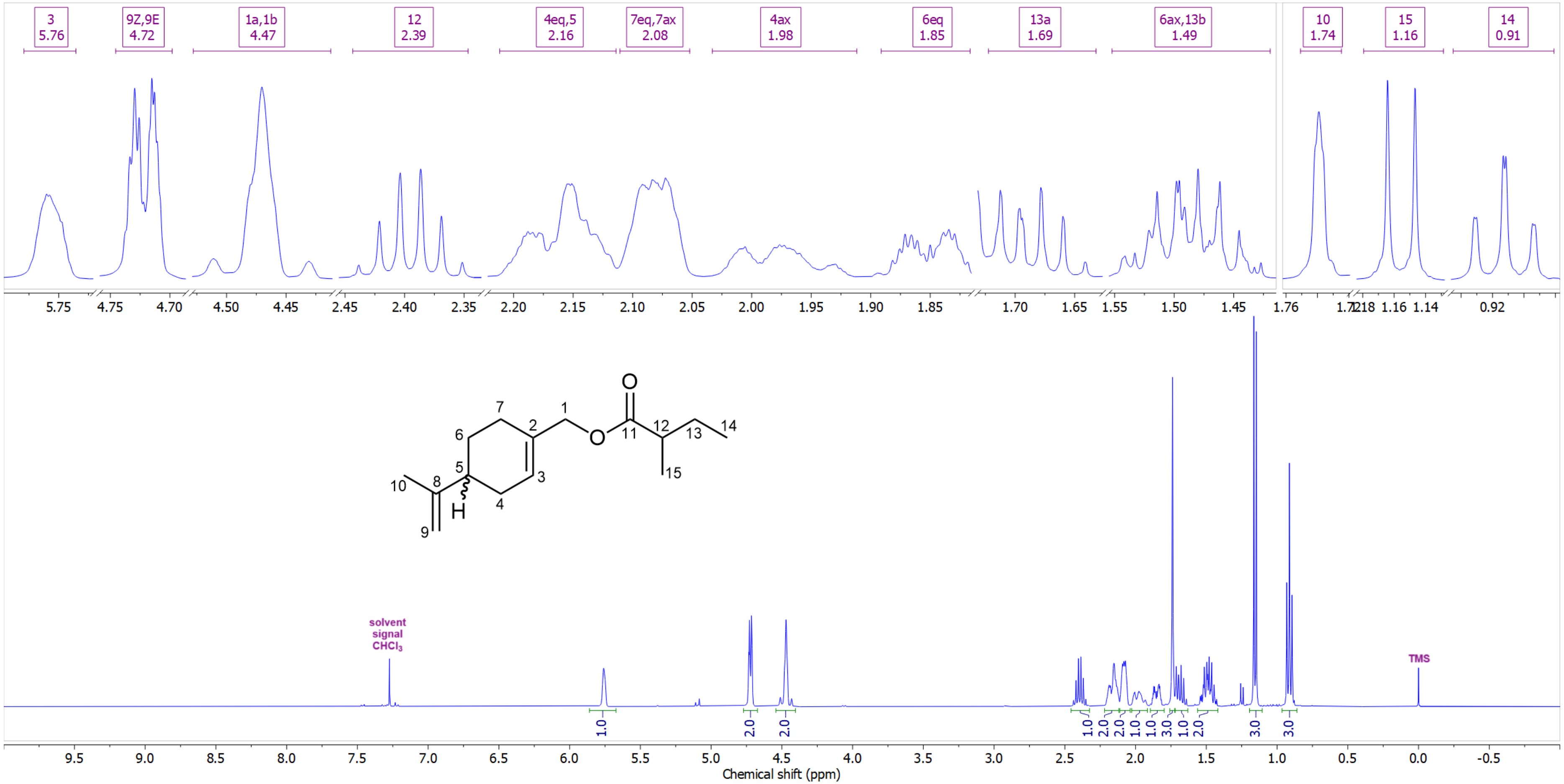

Figure S3. ^{1}H NMR (400 MHz, $CDCl_3$) spectrum of perillyl 2-methylbutanoate (diastereomer mixture)

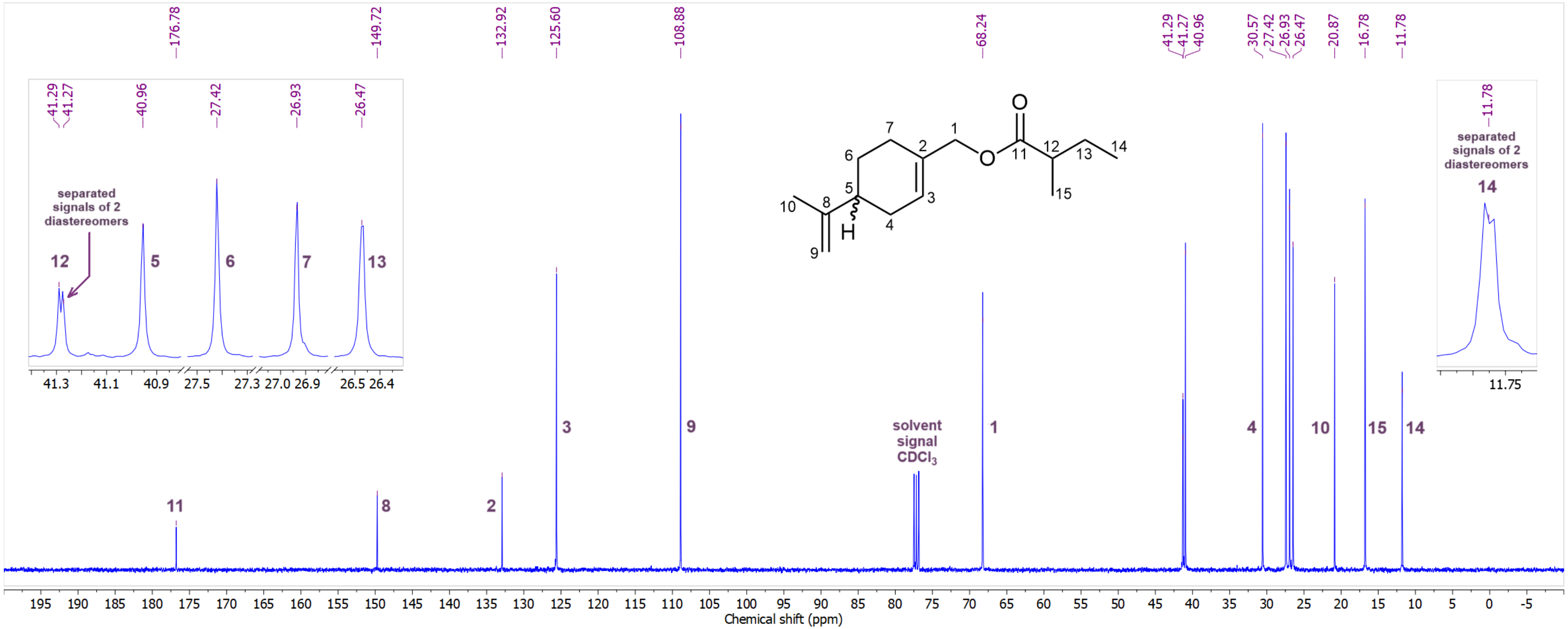

Figure S4. ^{13}C NMR (100.6 MHz, $CDCl_3$) spectrum of perillyl 2-methylbutanoate (diastereomer mixture)

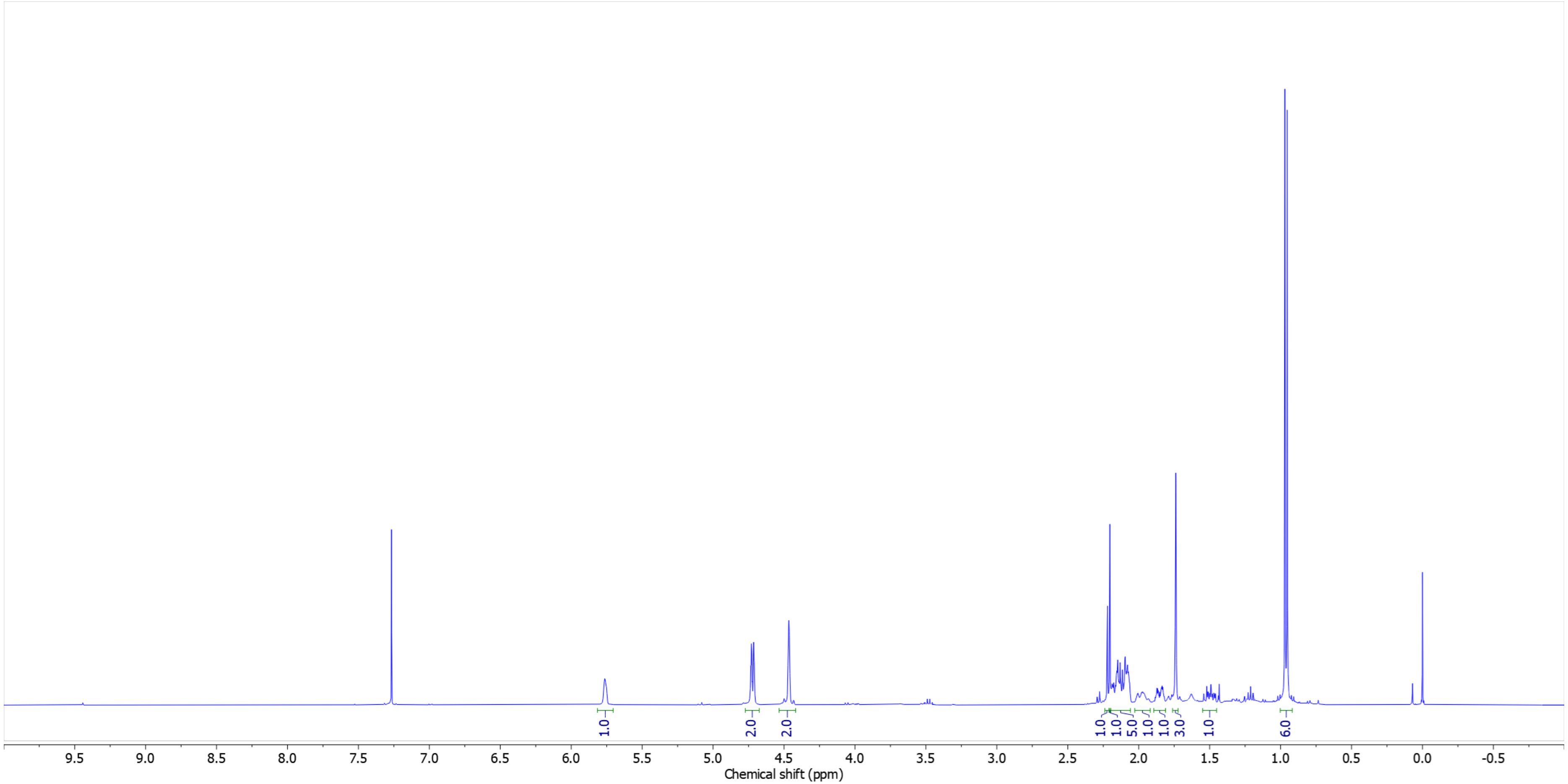

Figure S5. ^{1}H NMR (400 MHz, $CDCl_3$) spectrum of perillyl 3-methylbutanoate

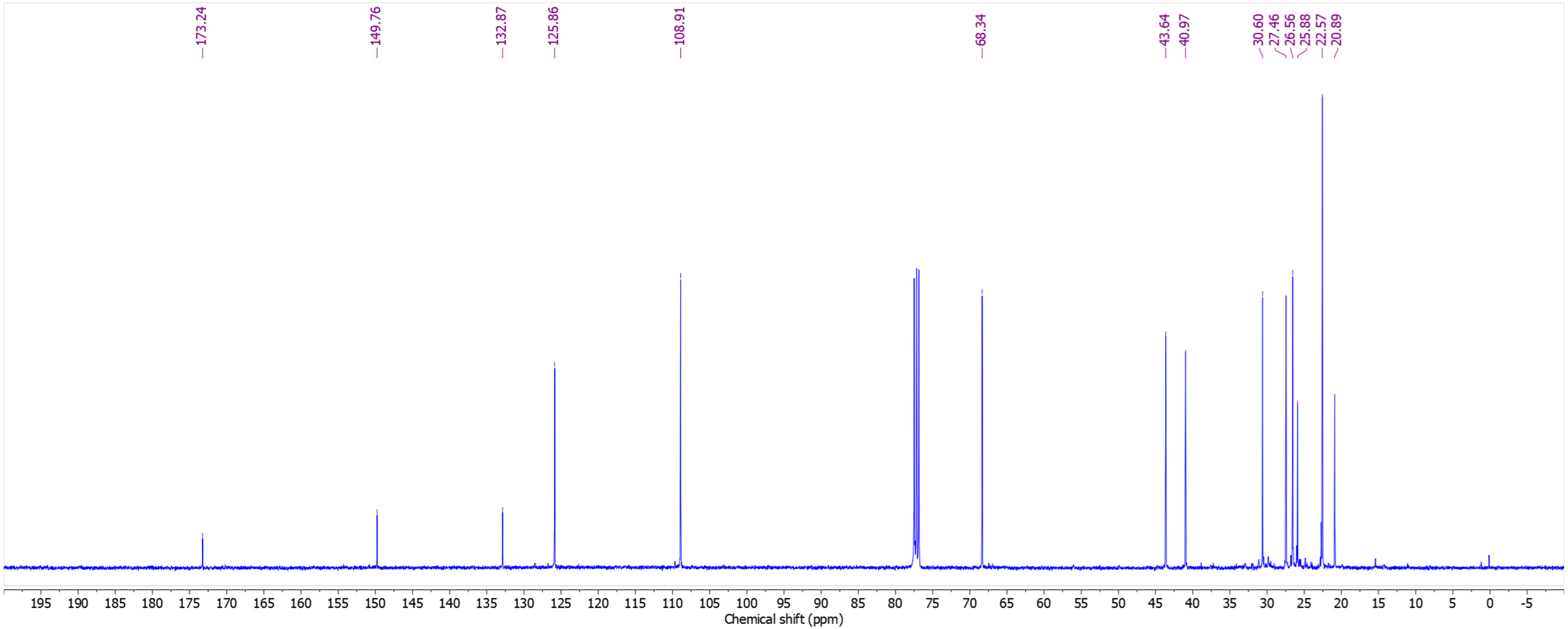

Figure S6. ^{13}C NMR (100.6 MHz, $CDCl_3$) spectrum of perillyl 3-methylbutanoate
